# Climate warming induced pervasive growth decline in Chinese pine populations of the Loess Plateau, China

**DOI:** 10.3389/fpls.2026.1749887

**Published:** 2026-02-24

**Authors:** Zongshan Li, Cong Wang, Guangyao Gao, Xiaoming Feng, Yihe Lv, Xiaochun Wang, Qindi Zhang, Haibin Liang, Xiaojuan Zhang, Yue Guo, Jianbo Liu

**Affiliations:** 1 State Key Laboratory of Regional and Urban Ecology, Research Center for Eco-Environmental Sciences, Chinese Academy of Sciences, Beijing, China; 2 Shaanxi Yan’an Forest Ecosystem National Observation and Research Station, Beijing, China; 3 National Observation and Research Station of Earth Critical Zone on the Loess Plateau in Shaanxi, Xi’an, China; 4 College of Forestry, Northeast Forestry University, Harbin, China; 5 College of Life Sciences, Shanxi Normal University, Taiyuan, China; 6 Institute of Geographical Science, Taiyuan Normal University, Jinzhong, China; 7 Ordos International Technological Innovation Center for Desertification Control, Ordos, China; 8 Tianjin Key Laboratory of Water Resources and Environment, Tianjin Normal University, Tianjin, China

**Keywords:** Chinese pine forests, climatic warming, radial growth, The Loess Plateau, tree rings

## Abstract

**Introduction:**

Modern warming and associated aridification have intensified forest growth decline and tree mortality, weakening forest carbon sequestration. This study aims to investigate how Chinese pine (*Pinus tabuliformis* Carr.) responds to these climatic shifts across the Loess Plateau, a region highly sensitive to environmental changes.

**Methods:**

We synthesized tree-ring width chronologies from 60 sites spanning major geomorphological units of the Loess Plateau. To evaluate the impact of rapid warming, we compared climate–growth relationships between two distinct periods: the pre-warming phase (1901–1960) and the post-warming phase (1961–2012).

**Results:**

Before 1960, when warming and drying were not pronounced, radial growth generally increased across regions, primarily limited by moisture while temperature had a modest stimulatory effect. After 1960, as warming and aridification strengthened, growth declined across all geomorphological units. Moisture limitation intensified, and higher temperatures shifted from a weak benefit to a clear suppressive influence. Spatially, the southern Loess Plateau showed the highest sensitivity to both growing- and non-growing-season climate, while the western and eastern regions were less sensitive and primarily influenced by non-growing-season conditions.

**Discussion:**

The spatially heterogeneous responses identified in this tree-ring dataset underscore the complex impact of recent warm–dry trends on forest ecosystems. These findings are crucial for improving our understanding of forest dynamics in semi-arid regions and can guide adaptive forest management strategies to sustain ecosystem functioning under ongoing aridification of the Loess Plateau.

## 1 Introduction

The Loess Plateau lies in the Asian monsoon region and is characterized by an arid to semi-arid climate (Lu et al., 2021). Its vegetation is fragile and highly sensitive to climate variability, especially water stress (Fu et al., 2022). Remote-sensing evidence indicates that recent climate warming has, to some extent, promoted vegetation growth, making the Plateau one of the most conspicuously greening regions in China and even worldwide (Lu et al., 2015; Li et al., 2018; Li et al., 2019). The beneficial effects of warming are expressed in two main ways. First, rising temperatures have increased vegetation growth rates and substantially improved regional vegetation cover (Feng et al., 2013; Jiang et al., 2021). Following the implementation of the Grain-for-Green program in 1999, mean vegetation cover in major Yellow River tributary basins increased from 29% in 1998 to 46% in 2010, and widespread greening has been reported across much of the Plateau (Jiao et al., 2010; Wang et al., 2016). This trend is commonly attributed to the combined influences of climate warming and large-scale ecological restoration (Wang et al., 2023a; Zha et al., 2025). Second, warming has been associated with increasing vegetation biomass and net primary productivity (NPP), thereby strengthening regional carbon sequestration capacity (Jin et al., 2017; Chen et al., 2021). For example, Feng et al. (2016) estimated a significant rise in total NPP in the Loess Plateau during 2000–2010 of 9.3 ± 1.3 gC m^-2^ yr^-1^. Overall, warming together with restoration initiatives has enhanced vegetation growth, cover, biomass, and carbon uptake on the Loess Plateau, with potential benefits for climate-change mitigation.

There remains considerable uncertainty regarding the persistence and stability of the positive impacts of climate warming on vegetation growth on the Loess Plateau (Fu et al., 2023; Wang et al., 2023a). On one hand, the Loess Plateau is a typical water-limited region, and large-scale vegetation restoration efforts may result in adverse effects such as soil desiccation and declining groundwater levels in certain areas (Zhang et al., 2019; Wu et al., 2021). Some studies suggest that the current level of vegetation cover on the Loess Plateau has already surpassed the equilibrium threshold that the regional climate can sustainably support (Feng et al., 2016). Excessive vegetation restoration has intensified competition for limited water resources, leading to soil moisture deficits. These conditions, in turn, constrain the long-term healthy growth of vegetation and threaten the sustainability of the ecosystem (Liang et al., 2019; Wang et al., 2020). On the other hand, vegetation on the Loess Plateau is predominantly artificial, with a relatively young average age. While climate warming has promoted vegetation growth in the short term, this growth acceleration is largely attributed to a pronounced juvenile effect. As the vegetation matures, the positive impact of climate warming on growth is expected to decline significantly (Song et al., 2022; Zhang et al., 2022). Moreover, although remote sensing data indicate an overall increase in vegetation greenness across the region, some studies suggest a concurrent decline in the ecological resilience of vegetation to external disturbances such as drought (Hu et al., 2023; Wang et al., 2023b). In the context of increasingly frequent and intense extreme climate events—such as droughts and heatwaves—the stability and sustainability of vegetation growth on the Loess Plateau are facing mounting challenges (Wang et al., 2024; Xu et al., 2024). As evident from current research, the impact of modern climate warming on vegetation growth on the Loess Plateau is complex and not entirely positive, with considerable uncertainty surrounding its long-term ecological consequences. To accurately assess the long-term effects of climate warming on regional vegetation, it is essential to examine the response characteristics of vegetation to climate change over extended temporal scales.

Due to their unique advantages, such as accurate dating, long time series, wide distribution, and clear climate signals, tree-ring materials have become important resources for studying forest growth responses to climate change within a long-term context (Cook et al., 2010; Zhang and Fang, 2020). The Loess Plateau lies in the transition zone between the monsoonal and non-monsoonal regions, where ecosystems are highly fragile and particularly sensitive to global climate change (Feng et al., 2016; Fu et al., 2022). In recent years, dendroclimatological and dendroecological studies in this region have attracted increasing attention. Based on tree-ring samples from multiple sites across the Loess Plateau, numerous studies have reconstructed precipitation, drought indices, and temperature variability over the past several centuries, providing valuable historical climate information for the region (Fang et al., 2010; Cai et al., 2015; Wang et al., 2017; Li et al., 2019; Chen et al., 2023; Hou et al., 2024). However, existing dendrochronological research on the Loess Plateau has been dominated by site-based climate reconstructions, whereas the spatial heterogeneity of forest growth responses to climate change at the regional scale has received comparatively limited attention (Liu et al., 2020; Chen and Han, 2023; Chen et al., 2024; Zhao et al., 2025). Given the recent tendency toward warmer and drier conditions on the Loess Plateau, spatially explicit tree-ring datasets are essential for robustly characterizing the interannual growth responses and adaptive strategies of regional forests under increasing aridity (Choat et al., 2012; Anderegg et al., 2022; Cabon et al., 2022).

Chinese pine (*Pinus tabulaeformis* Carr.) is a dominant species of the zonal forest vegetation on the Loess Plateau and is widely distributed across the region (Chen et al., 2023). Broadleaf forests at low elevation zone on the Loess Plateau have markedly contracted due to intensive human activities since early 20^th^ century, whereas Chinese pine forests—occurring predominantly in higher-elevation mountainous areas—have experienced relatively limited human disturbance thus have thus been preserved in a comparatively intact state (Li et al., 2016; Qin et al., 2017). Therefore, at the regional scale, radial growth of Chinese pine and its climatic responses can serve as a representative indicator of overall forest growth trends and the sensitivity of forest vegetation to climate change across the Loess Plateau. This study integrates tree-ring data of Chinese pine forests from different geomorphic units of the Loess Plateau, including southern Loess Plateau (Qinling Mountains and its adjacent mountainous areas), eastern Loess Plateau (the Taihang and Lüliang Mountains) and western Loess Plateau (the northeastern margin of the Tibetan Plateau and the Henan Mountain). The aim is to reveal the long-term response characteristics and spatial heterogeneity of vegetation growth to climate warming in the Loess Plateau region. We propose the following two research hypotheses: 1) The recent warming‐induced intensification of drought has increasingly constrained the radial growth of Chinese pine forests across the Loess Plateau. 2) The drought impact on the radial growth of these forests exhibits pronounced spatial heterogeneity.

## 2 Material and methods

### 2.1 Tree ring data

**Figure 1:**
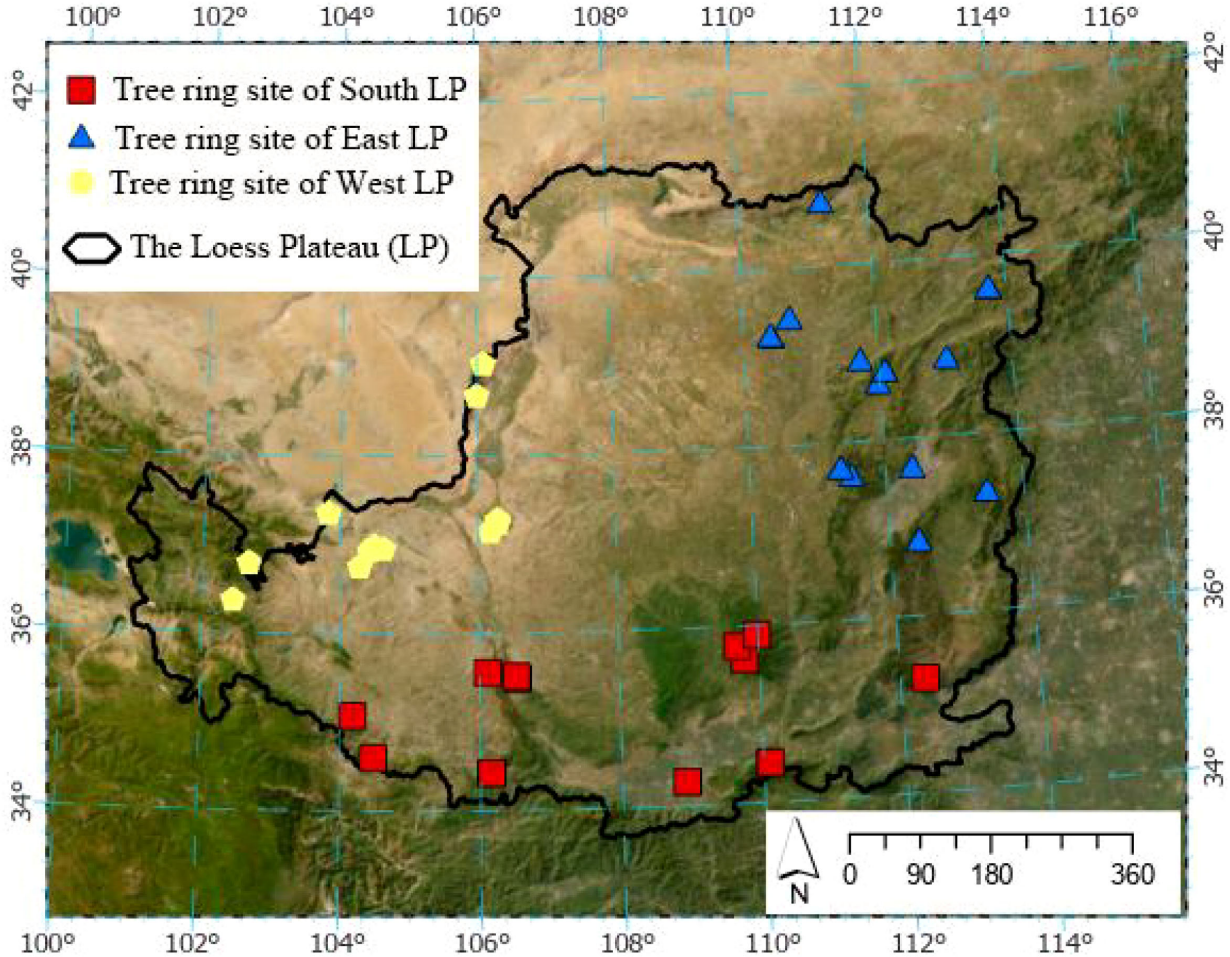
Locations of tree-ring chronologies in Chinese pine forests of the Loess Plateau, China. South LP, East LP and West LP indicate southern Loess Plateau (Qinling Mountains and its adjacent mountainous areas), eastern Loess Plateau (the Taihang and Lüliang Mountains) and western Loess Plateau (the northeastern margin of the Tibetan Plateau and the Henan Mountain), respectively. The base map was the Esri global online orthophoto imagery (15 m spatial resolution), which was used to highlight the major geographical and geomorphological features of the Loess Plateau.

This study compiled a tree-ring dataset of 60 sample sites for *P. tabuliformis* forests from the Loess Plateau, and all those tree-ring chronology data were retrieved from published literature (**Figure 1**; **Supplementary Table S1**). We performed a Boolean search using alternative combinations with the following keywords: “tree rings”, “radial growth”, “climate-response” and “climate-reconstruction” to survey tree-ring related studies located at northeastern China in Web of Science, Google Scholar and the China National Knowledge Infrastructure (https://oversea.cnki.net/index/). Tree-ring data were digitalized directly from the figures of tree-ring chronologies or reconstruction series from the targeted literature by GetData software (v.2.26; http://getdata-graph-digitizer.com/) (Mu et al., 2023). Those tree-ring data from literature were further filtered with the ‘cubic smoothing spline’ approach, with a 50% frequency-response cutoff at 30 years to remove low-frequency fluctuations in chronologies (Cook and Kairiukstis, 1990). This method effectively mitigates the potential influence of factors such as chronology types and detrending methods in different sources of literature on tree-ring data outcomes. Consequently, the extracted tree-ring data series exhibit similar mean values and distribution characteristics, thereby facilitating more robust comparative analysis between them. Chronologies of tree-ring widths retrieved in this study based on the following criteria: availability of metadata (longitude, latitude, elevation and species name), tree ring series covering at least 90 years (from 1901 to 1990) between 1901 and 2012, and availability of tree-ring width measurements or tree-ring width chronologies.

Based on the spatial distribution of the tree-ring chronologies across the Loess Plateau (**Figure 1**; **Supplementary Table S1**), we broadly divided the dataset into three subregions: the southern Loess Plateau (the Qinling Mountains and adjacent mountainous areas, 20 sample sites), the eastern Loess Plateau (the Taihang and Lüliang Mountains, 19 sample sites), and the western Loess Plateau (the northeastern Tibetan Plateau and the Helan Mountain, 21 sample sites). These subregions were delineated primarily according to the major physiographic and geomorphological features of the Loess Plateau.

### 2.2 Climate data and statistical analysis

**Figure 2:**
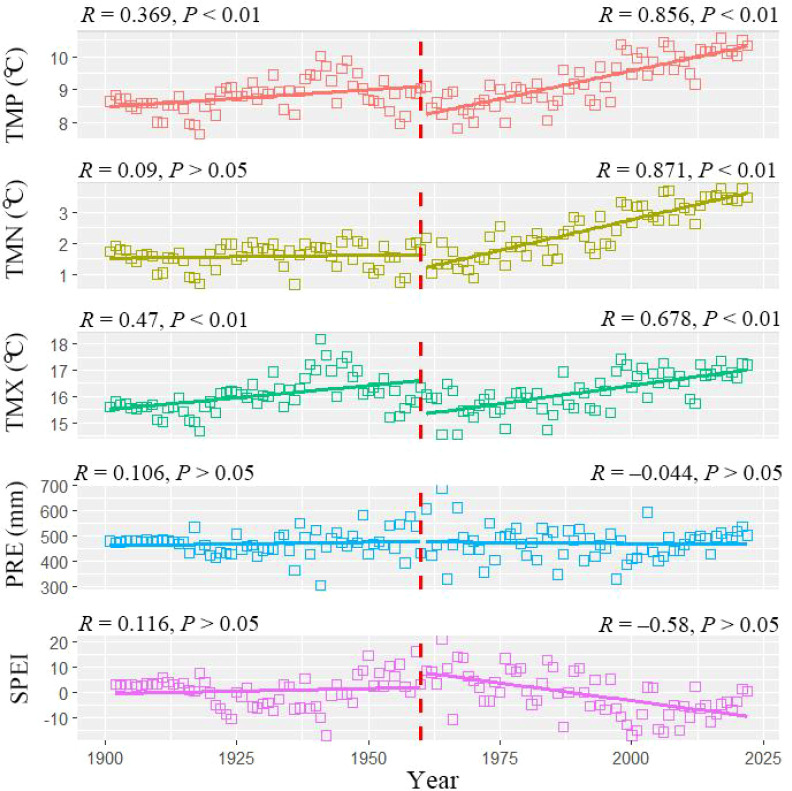
Comparison of changing trends during two distinct periods (1901–1960 and 1961–2012) for climatic variables in the Loess Plateau, China. TMP, TMN, TMX, PRE, SPEI indicated mean temperature, minimum temperature, maximum temperature, total precipitation and standardized precipitation evapotranspiration index, respectively. *R* indicates the correlation coefficients between timeseries and climate data, and the red bold vertical dashed line indicates the year (1960) that separates the two analysis periods. .

We extracted climate data of the 60 tree-ring sites in this study from the nearby gridded climate data from the CRU TS v.4.07 global climate dataset. The CRU TS v.4.07 dataset (https://crudata.uea.ac.uk/cru/data/hrg/cru_ts_4.07/) (Harris et al., 2014) had a spatial resolution of 0.5°(approximately 50 km). The CRU data covered the period from 1901 to 2020 and included five climatic elements: monthly mean temperature (TMP), maximum temperature (TMX), minimum temperature (TMN), annual total precipitation (PRE), and the standardized precipitation evapotranspiration index (SPEI). Because data for different temperature variables consistently demonstrate that the climate in the study area has experienced a marked and rapid warming trend since approximately 1960 (**Figure 2**), the correlation analysis between tree ring data and climate variables was conducted for two distinct time periods (1901–1960 and 1961–2012 periods) using the DendroClim 2002 software (Biondi and Waikul, 2004). Considering the influence of previous year’s climate factors on current-year tree growth, we used climate data from June of the previous year to October of the current year for the correlation analysis with the standardized tree-ring chronology (Fritts, 1966).

## 3 Results

### 3.1 The comparison of the trends in climate variables before and after climatic warming

Before 1960 (during the period 1901–1960), climate warming on the Loess Plateau was not significant, and the overall climate exhibited a warm and humid trend (**Figure 2**). Specifically, the interannual mean and maximum temperatures showed a certain upward trend (*R* = 0.369–0.47, *P* > 0.01), while the interannual minimum temperature showed no significant trend (*R* = 0.09, *P* > 0.05). In addition, both the annual total precipitation (*R* = 0.283, *P* < 0.05) and the annual average SPEI drought index (*R* = 0.106–0.116, *P* > 0.05) exhibited certain increasing trends, although the latter did not reach statistical significance. After 1960 (during the period 1961–2012), the study area experienced significant climate warming and exhibited an overall warm-dry climate trend (**Figure 2**). Specifically, the interannual mean, minimum, and maximum temperatures all showed significant upward trends (*R* = 0.678–0.871, *P* < 0.01). In contrast, the annual average SPEI drought index showed a significant downward trend (*R* = –0.58, *P* < 0.05), and the annual total precipitation exhibited a decreasing trend as well (*R* = –0.044, *P* > 0.05), although the latter was not statistically significant.

The characteristics of climate change in different geomorphic units of the Loess Plateau (including the Qinling Mountains and surrounding mountainous areas, the Taihang and Lüliang Mountains, and the northeastern Tibetan Plateau and the Helan Mountain) are largely consistent with the overall trends observed across the Loess Plateau (**Supplementary Figures S1**-**S3**). Specifically, during the period before 1960 (1901–1960), these regions exhibited a certain warming trend, while both precipitation and the SPEI drought index showed slight upward trends, indicating an overall shift toward a warm and humid climate. After 1960 (1961–2012), a significant warming trend was observed, accompanied by a clear decline in the SPEI drought index, reflecting an overall shift toward a warm and dry climate.

### 3.2 The comparison of the trends in tree growth before and after climatic warming

The tree-ring chronologies of Chinese pine forests from different geomorphic units of the Loess Plateau exhibit strong positive correlations within their common time intervals (**Supplementary Figures S1**–**S3**). This indicates a high level of consistency in the interannual variability of radial growth of Chinese pine across different locations and geomorphic units. Therefore, the averaged chronology index can be considered representative of the interannual variability in radial growth of Chinese pine across the various geomorphic units of the Loess Plateau.

**Figure 3:**
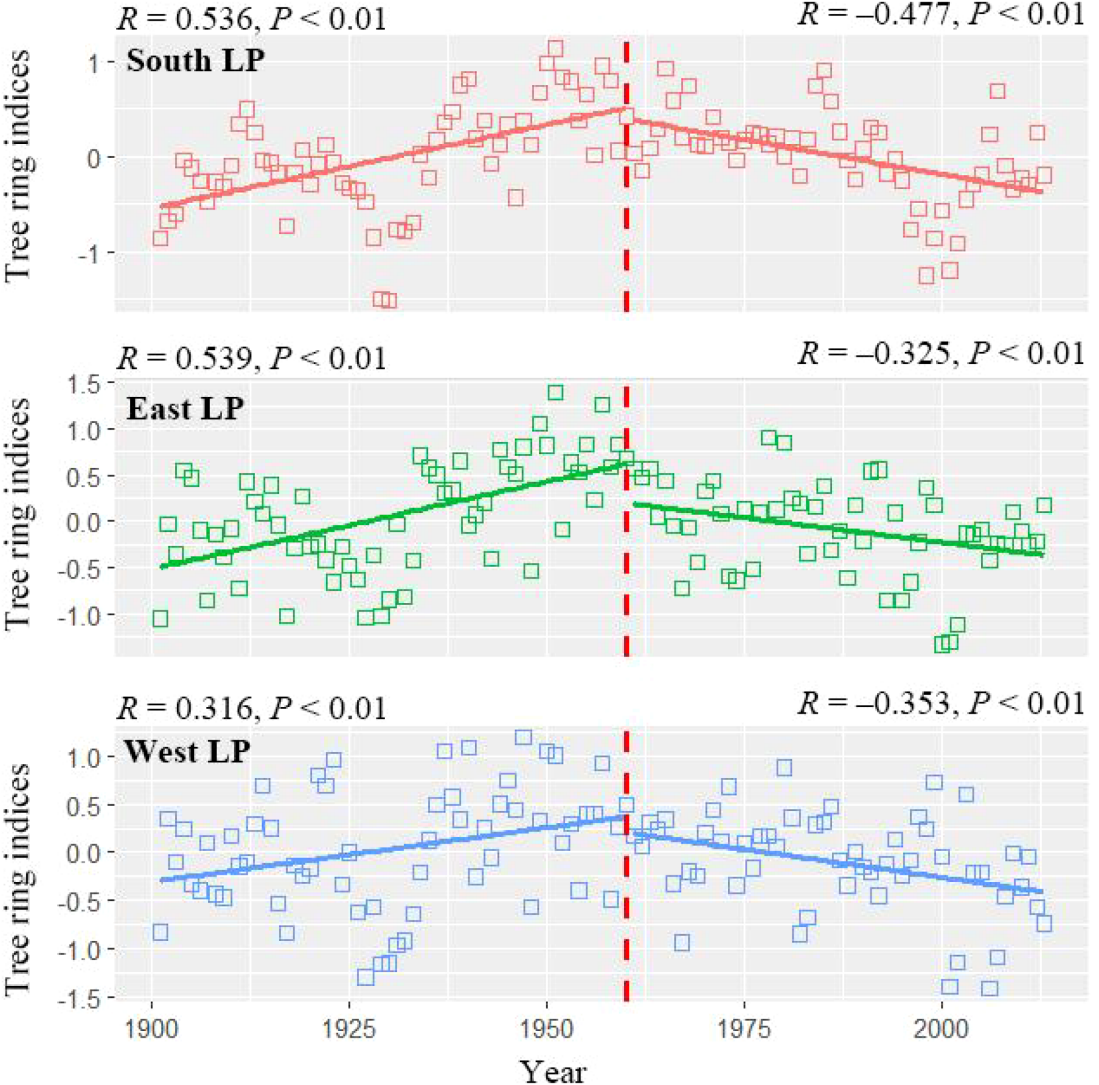
Comparison of growth trends during two distinct periods (1901–1960 and 1961–2012) for Chinese pine forests of the Loess Plateau, China. South LP, East LP and West LP indicated the southern part (Qinling and nearby mountainous areas), eastern part (Taihang and Lüliang mountain ranges) and western part (the northeastern edge of the Tibetan Plateau and the Helan Mountain) of the Loess plateau, respectively. *R* indicates the correlation coefficients between timeseries and tree ring data, and the red bold vertical dashed line indicates the year (1960) that separates the two analysis periods.

The mean chronologies of Chinese pine from different geomorphic units of the Loess Plateau exhibit distinctly different growth trends before and after the onset of climate warming (i.e., during the periods 1901–1960 and 1961–2012, respectively) (**Figure 3**). Prior to climate warming, the tree-ring indices of Chinese pine in all geomorphic units showed a significant upward trend, with the most pronounced increases observed in the southern Loess Plateau, as well as the eastern Loess Plateau (*R* = 0.536–0.539, *P* < 0.01), followed by the northeastern edge of the Qinghai–Tibet Plateau (*R* = 0.316, *P* < 0.01). However, after the onset of climate warming, the tree-ring indices of Chinese pine in all geomorphic units exhibited a significant downward trend, with the most notable decline in the southern Loess Plateau (*R* = –0.477, *P* < 0.01), followed by the eastern and western Loess Plateau (*R* = –0.325 to –0.353, *P* < 0.01).

### 3.3 Comparative characteristics of tree growth sensitivity to climate before and after climate warming

**Figure 4:**
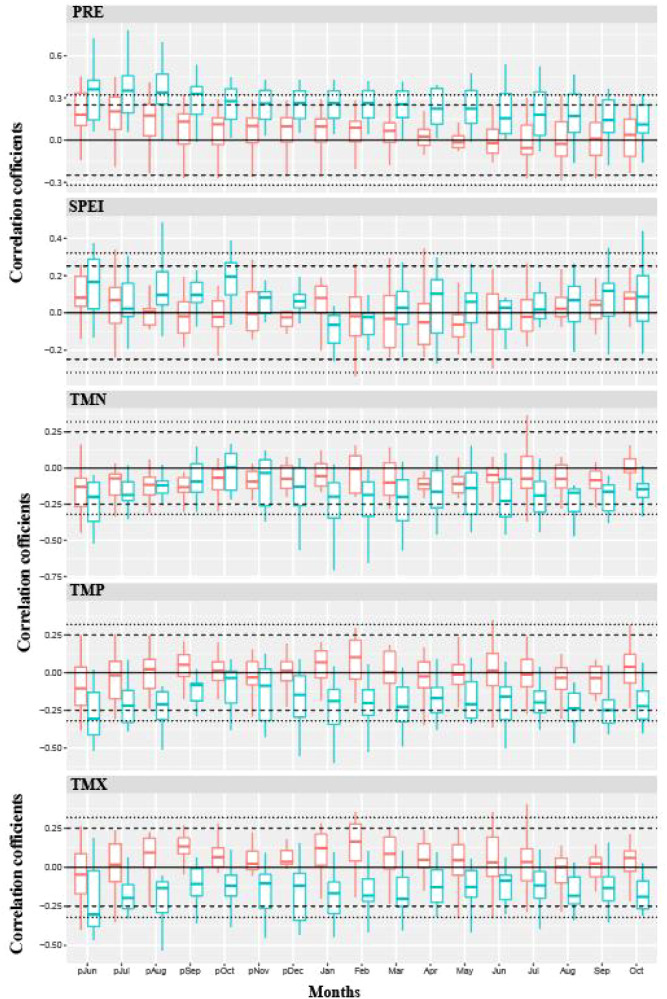
The correlations between climate variables and tree-ring chronologies of Chinese pine forests during two distinct periods (1901–1960 and 1961–2012) in the southern Loess Plateau (the Qinling and surrounding mountainous areas). TMP, TMN, TMX, PRE, SPEI indicated mean temperature, minimum temperature, maximum temperature, total precipitation and standardized precipitation evapotranspiration index, respectively. The red and green bars indicated the correlation analysis for 1901–1960 and 1961–2012 period, respectively. The dotted and dashed lines indicated correlation levels reach 95% and 99% significance, respectively.

**Figure 5:**
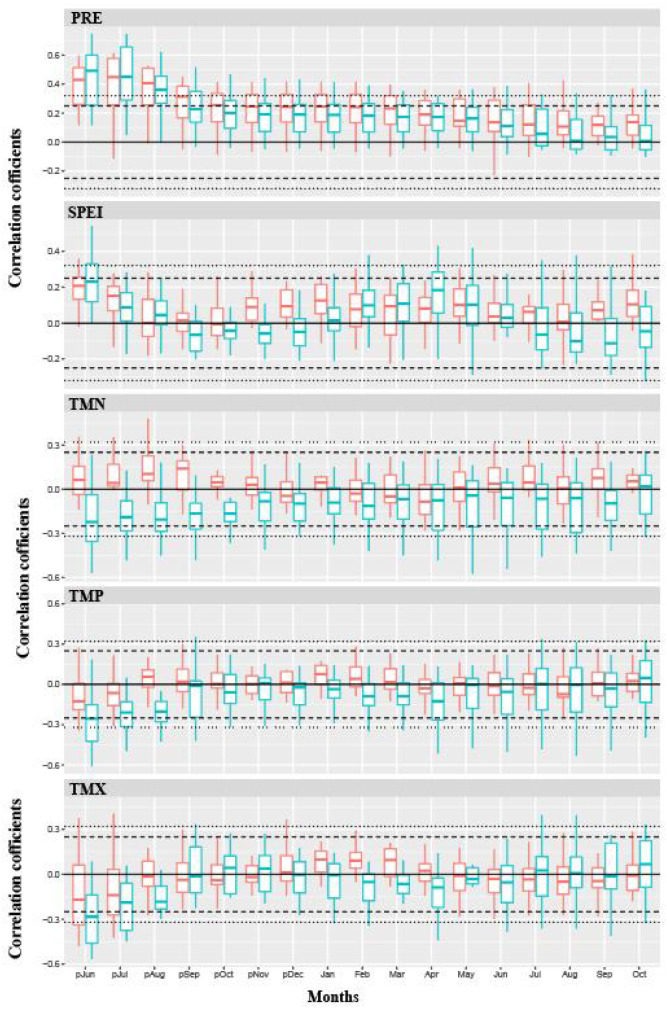
The correlations between climate variables and tree-ring chronologies of Chinese pine forests during two distinct periods (1901–1960 and 1961–2012) in eastern Loess Plateau (the Taihang and Lüliang mountainous areas). TMP, TMN, TMX, PRE, SPEI indicated mean temperature, minimum temperature, maximum temperature, total precipitation and standardized precipitation evapotranspiration index, respectively. The red and green bars indicated the correlation analysis for 1901–1960 and 1961–2012 period, respectively. The dotted and dashed lines indicated correlation levels reach 95% and 99% significance, respectively.

**Figure 6:**
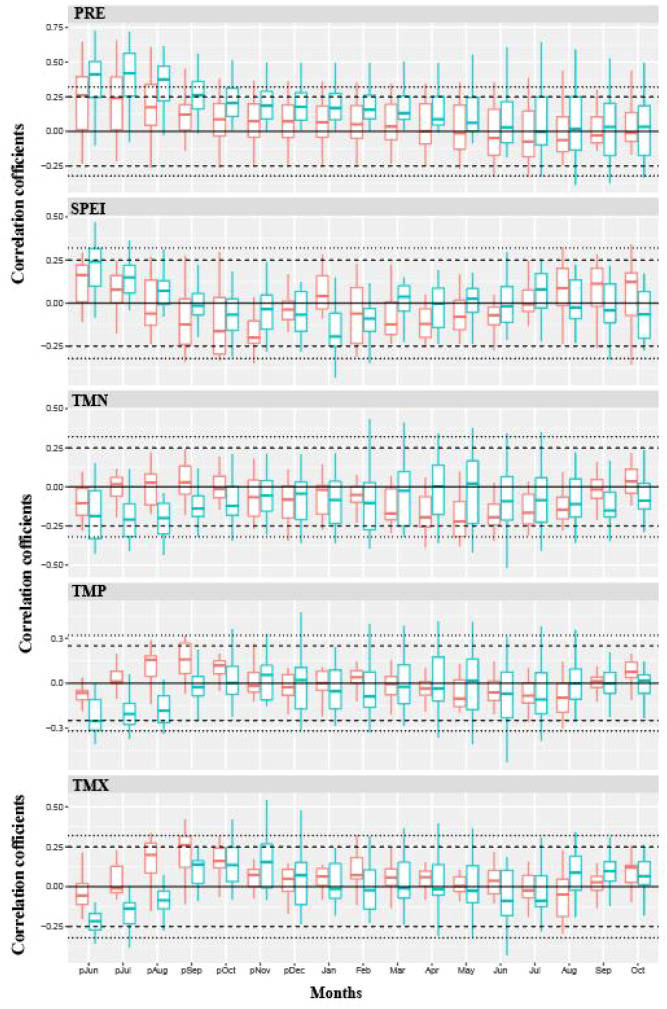
The correlations between climate variables and tree-ring chronologies of Chinese pine forests during two distinct periods (1901–1960 and 1961–2012) in western Loess Plateau (the northeastern Tibetan Plateau and the Helan Mountain). TMP, TMN, TMX, PRE, SPEI indicated mean temperature, minimum temperature, maximum temperature, total precipitation and standardized precipitation evapotranspiration index, respectively. The red and green bars indicated the correlation analysis for 1901–1960 and 1961–2012 period, respectively. The dotted and dashed lines indicated correlation levels reach 95% and 99% significance, respectively.

Based on the response analysis of the chronologies of Chinese pine to climatic factors in different geomorphic units of the Loess Plateau (**Figures 4**–**6**), there are significant differences in climatic sensitivity of the chronologies before and after the climate warming around 1960. In the southern Loess Plateau, the correlations between Chinese pine chronologies and various temperature variables were relatively weak before 1960. During that period, the chronologies showed only a certain positive correlation with the maximum temperatures in the previous winter and the current spring. However, after the climate warming around 1960, the negative correlations between the chronology and temperature variables significantly increased, and the negative correlations for most months reached statistically significant levels (**Figure 4**). Before 1960, the correlation between the Chinese pine chronology in the southern Loess Plateau and precipitation was also relatively weak, showing a clear positive correlation only with the previous summer’s precipitation. After 1960, however, the positive correlation between the chronology and precipitation significantly strengthened, with most monthly correlations reaching statistically significant levels. In contrast, the correlation between the chronology and the SPEI drought indices remained weak both before and after the climate warming around 1960, with no significant differences observed during the two periods.

Before the climate warming in 1960, the correlations between Chinese pine chronologies of the eastern Loess Plateau and temperature factors was relatively weak (**Figure 5**). It was only reflected in a certain negative correlations with the previous summer’s temperature factors, with the negative correlation being significant only with the maximum temperature of the previous summer. However, after the climate warming in 1960, the correlations with temperature factors became significantly stronger, mainly manifested in a significant negative correlation with the previous summer’s temperature factors. In addition, the chronologies also showed a noticeable negative correlation with the minimum temperature during the current growing season. Furthermore, the chronologies has generally maintained a positive correlation with precipitation both before and after the climate warming in 1960, with the most significant positive correlation being with the previous summer’s precipitation. The correlation with the SPEI drought index was relatively weak before and after 1960, with a certain positive correlation only observed with the previous summer.

Before the climate warming in 1960, Chinese pine chronologies on the western Loess Plateau showed a strong positive correlation with the previous summer’s temperature (**Figure 6**). However, after the climate warming in 1960, the chronologies shifted to a strong negative correlation with the previous summer’s temperature. Additionally, before 1960, the tree-ring chronology exhibited a certain negative correlation with the spring temperature of the same year, but after 1960, the relationship between the tree-ring chronology and the spring temperature became weaker. The chronologies maintained a clear positive correlation with the previous summer’s precipitation and SPEI drought index both before and after the 1960 climate warming, with the strength of this positive correlation significantly increasing after the climate warming in 1960.

## 4 Discussion

### 4.1 The overall trend of the response of radial growth of Chinese pine forest to climate warming in the Loess Plateau region

The trend of radial growth and climate sensitivity of Chinese pine in the Loess Plateau region exhibits distinct contrasting characteristics before and after the climate warming of the time of 1960. Prior to this significant warming, the temperature increase in the Loess Plateau was relatively minor, and the climate was generally warmer and wetter. During this period, the radial growth of Chinese pine across various geomorphological units showed a noticeable upward trend. The chronologies of Chinese pine maintained positive correlations with both water availability and temperature factors, indicating that the overall warmer and wetter climate promoted the radial growth of this species. Before 1960, the average temperature was relatively low with a minor increase. Within suitable ranges, rising temperatures could benefit the temperature accumulation in spring and delay the cooling process in autumn, thereby extending the photosynthesis period of Chinese pine. This extension promoted the accumulation of organic matter, ultimately resulting in increased radial growth (Myneni et al., 1997; Briffa et al., 1998). Additionally, the relatively plentiful precipitation before 1960 could directly alleviate potential water stress encountered by Chinese pine during growth, ensuring its normal physiological activities, including photosynthesis, nutrient absorption and transport. This, in turn, promoted cell division and xylem formation, leading to an increase in radial growth (Liu et al., 2020; Chen et al., 2024).

After the significant climate warming in 1960, the Loess Plateau region experienced a larger degree of warming, and the climate generally showed warmer and drier characteristics. In this period, the radial growth of Chinese pine*s* in various geomorphological units of the Loess Plateau showed a clear downward trend. The positive correlation between the tree-ring chronologies of Chinese pine and water conditions, as well as the negative correlation with temperature factors, were both further strengthened, indicating that the warmer and drier climate overall suppressed the radial growth of Chinese pine.

After 1960, the rapid increase in temperature led to enhanced transpiration in plants and rapid loss of soil moisture, exacerbating the negative impact of water stress on the growth of Chinese pine (Zhao et al., 2021; Yue et al., 2024). Additionally, the fast rate of warming may exceed the physiological tolerance threshold of Chinese pine, resulting in decreased photosynthetic efficiency, increased respiratory consumption, and thermal damage to important physiological molecules such as proteins, ultimately suppressing tree growth (Cabon et al., 2019; Hou et al., 2024). The warming and drying climate conditions caused Chinese pine to close its stomata to reduce water loss in response to drought stress (Cabon et al., 2022; Shen et al., 2024). However, the closure of stomata also limits carbon dioxide absorption, thereby inhibiting photosynthesis, reducing organic matter synthesis, and ultimately leading to a slowdown in radial growth (Liu et al., 2019a; McDowell et al., 2022). Furthermore, the warming and drying conditions favor the reproduction and spread of pests and diseases, further threatening the health of Chinese pine and indirectly suppressing its growth (Choat et al., 2012; Li et al., 2022).

### 4.2 The spatial heterogeneity characteristics of the response of radial growth of Chinese pine to climate warming in the Loess Plateau region

The response sensitivity characteristics of radial growth of Chinese pine to drought stress caused by recent climate warming exhibit significant differences across various geomorphological units of the Loess Plateau. In the southern Loess Plateau, the radial growth of Chinese pine is highly sensitive to drought stress, with both current and previous year’s drought conditions having a pronounced inhibitory effect on tree growth. In contrast, in the eastern and western Loess Plateau, the sensitivity of radial growth of Chinese pine to drought stress induced by recent climate warming is relatively low, manifesting primarily as a significant inhibitory effect on tree growth from last summer’s drought stress.

The Qinling Mountains and nearby mountainous areas are located in the southern part of the Loess Plateau, where the climate conditions are relatively favorable. As a result, the tolerance of Chinese pine to drought stress is relatively low. Factors such as shallower root depth, lower water-use efficiency, and weaker osmotic regulation make it challenging for the trees to maintain basic physiological activities under drought stress, leading to a high sensitivity to both current and previous year’s drought conditions (Kerr et al., 2023; Senf et al., 2025). In contrast, the eastern Loess Plateau (the Taihang and Lüliang Mountains) and the western Loess Plateau (the northeastern edge of the Tibetan Plateau and Helan Mountain) are situated in the northern part of China, where the climate conditions are relatively harsh. Consequently, the tolerance of Chinese pine to drought stress is higher in these areas. Factors such as deeper root depth, higher water-use efficiency, and stronger osmotic regulation enable these trees to maintain essential physiological activities even under drought stress, resulting in a high sensitivity only to last summer’s drought conditions (Anderegg et al., 2022; Quetin et al., 2024).

The coupling relationship between the radial growth of Chinese pine and last summer’s drought stress is ecologically significant. On one hand, last summer may have coincided with a critical period for the radial growth of Chinese pine, during which water deficiency directly limits cell expansion and nutrient accumulation in the xylem, subsequently affecting the carbohydrate reserves and physiological preparations needed for growth in the following year. This, in turn, significantly impacts the radial growth amount during the current growing season (Cai et al., 2015; Tardif et al., 2003). On the other hand, last summer’s drought stress adversely affects the non-growing season dormancy physiology of Chinese pine, such as increasing respiratory consumption and reducing cold resistance, thereby influencing the onset and growth rate of the current growing season (Liu et al., 2019b; Treydte et al., 2006).

### 4.3 Implications for the protection and restoration of the pine forest vegetation in the Loess Plateau

The sensitivity characteristics of the response of radial growth of Chinese pine to recent warming and drying climate vary significantly across different geomorphological units of the Loess Plateau, which will have important implications for the protection and restoration of Chinese pine forest vegetation in this region. In the southern Loess Plateau, Chinese pine exhibits a higher sensitivity to drought stress, and under the ongoing warming and drying climate, the forests in this area may face an increased risk of forest growth decline and tree mortality (Choat et al., 2012; Anderegg et al., 2022). Strict conservation measures should be implemented for Chinese pine forests in this region, such as limiting human disturbances (overharvesting, cultivation, etc.), enhancing monitoring and control of pests and diseases, and improving the natural recovery capacity of existing forests (Li et al., 2022; McDowell et al., 2022). The southern Loess Plateau is often important water sources, and the protection and restoration of vegetation, particularly forest types with strong water conservation capacity, may necessitate considering mixed forests rather than pure Chinese pine forests, which is significant for alleviating regional drought (Jactel et al., 2017; Messier et al., 2019). For Chinese pine forests in the southern Loess Plateau that exhibit growth decline characteristics, appropriate thinning should be conducted to reduce stand density, decrease competition for water among individual trees, and enhance the drought resistance of the remaining trees (D’Amato et al., 2011; Keenan, 2015).

In the eastern and western Loess Plateau, Chinese pine exhibits lower sensitivity to drought stress, indicating that it has strong environmental adaptability in these regions of the Loess Plateau, where the climate conditions are relatively harsh. Chinese pine forests play an important role in maintaining the basic functions of the local forest ecosystem and have high ecological protection and restoration value (Liu et al., 2020; Chen et al., 2024). Water management should focus more on enhancing soil water retention capacity during the growing season of Chinese pine to ensure sufficient water supply for normal growth and development during this period (Cai et al., 2015). When conducting artificial restoration in the northeastern edge of the Tibetan Plateau, it is essential to fully consider the local specific environmental conditions and select tree species that can adapt to high altitude, low temperatures, and certain drought stress (Briffa et al., 1998; Treydte et al., 2006). Additionally, factors such as non-growing season snowfall and soil freeze-thaw cycles, which significantly impact water supply and vegetation growth in high-altitude areas, should be incorporated into forest management and restoration considerations (Fritts, 1966; Vaganov et al., 1999).

## 5 Conclusion

This study found that in the Loess Plateau region, the radial growth of Chinese pine exhibited a significant upward trend before the notable climate warming in 1960, during which the warming amplitude was small and the overall climate was characterized by warmth and humidity. The warm and humid climate of this period generally promoted the radial growth of Chinese pine forest across different geomorphological units in the Loess Plateau. After 1960, with a substantial increase in warming and an overall trend towards a warmer and drier climate, the radial growth of Chinese pine in various geomorphological units of the Loess Plateau showed a significant downward trend, indicating that the warmer and drier climate generally suppressed the radial growth of Chinese pine. There were notable differences in the sensitivity of radial growth to modern warming and drying climate conditions across different geomorphological units in the Loess Plateau. In the southern Loess Plateau, the radial growth of Chinese pine showed high sensitivity to the warming and drying climate, with both current and previous year’s drought stress having a significant inhibitory effect on tree growth. In contrast, in the eastern and western Loess Plateau, the sensitivity of radial growth of Chinese pine to the warming and drying climate was lower, primarily reflecting a significant inhibitory effect on tree growth from last summer’s drought stress. This study reveals the spatial heterogeneity characteristics of the response of Chinese pine in different geomorphological units of the Loess Plateau to modern warming and drying climate, providing a scientific basis for formulating precise and regional forest vegetation protection and restoration strategies of for Chinese pine forests the Loess Plateau.

## Data Availability

The original contributions presented in the study are included in the article/**Supplementary Material**, further inquiries can be directed to the corresponding author/s.
