# Supplementary material for: Climate warming induced pervasive growth decline in Chinese pine populations of the Loess Plateau, China
Source: Front Plant Sci. 2026 Feb 24;17:1749887. doi: 10.3389/fpls.2026.1749887 (PMC12971888; doi:10.3389/fpls.2026.1749887)
Supplement: Supplementary file 1 [file Supplementaryfile1.pdf]

Supplementary Material

Table S1 Information about tree-ring chronologies in Chinese pine forests of the Loess Plateau, China

Region	Longitude	Latitude	PRE (mm)	TEM (°C)	ALT (m)	Refs cited
West LP	106.08	39.08	50		1800	Ma, 2003
West LP	102.65	36.83	436.5	2.25	2200	Li *et al.*, 2012
West LP	106.27	37.32	240.6	9.2	2400	Wang *et al.*, 2010
West LP	103.8	37.4	306.7	4.9	2500	Gao *et al.*, 2005
West LP	105.98	38.72	20	10	2200	Li *et al.*, 2007
West LP	106.08	39.08	20	10	2200	Li *et al.*, 2007
West LP	106.27	37.3			2400	Lu *et al.*, 2013
West LP	106.27	37.32	240.6	9.2	2300	Wang *et al.*, 2013
West LP	106.27	37.3	240.6	9.2	2400	Wang *et al.*, 2013
West LP	106.27	37.3	240.6	9.2	2400	Wang *et al.*, 2013
West LP	106.27	37.28	240.6	9.2	2600	Wang *et al.*, 2013
West LP	106.27	37.3	240.6	9.2	2400	Wang *et al.*, 2013
West LP	106.08	39.08			2600	Zhang *et al.*, 2009
West LP	105.98	38.72			2400	Zhang *et al.*, 2009
West LP	104.4	36.98			2600	Zhang *et al.*, 2009
West LP	104.47	37.03			2600	Kang *et al.*, 2012
West LP	104.6	37			2600	Ma *et al.*, 2015
West LP	102.43	36.41				Wang *et al.*, 2009a

West LP	104.26	36.8	380	6.5	2708.5	Jia *et al*., 2014
West LP	106.16	37.19	271.9	8.9	2300	Wang *et al*., 2009c
West LP	106.16	37.19	271.9	8.9	2300	Wang *et al*., 2009c
South LP	109.97	35.92	611.8	8.6	1370	Cai *et al*., 2008
South LP	110.07	34.47	900	5.9	2082	Liu *et al*., 2009a
South LP	108.9	34.3	800	14	1550	Liu *et al*., 2009b
South LP	108.9	34.3	800	14	2100	Liu *et al*., 2009b
South LP	109.78	35.65	611.8	8.6	1427	Cai *et al*., 2005
South LP	106.13	34.45	518.5	10.9	2660	Fang *et al*., 2012a
South LP	106.53	35.52	503	8.8	2000	Song *et al*., 2011
South LP	106.51	35.54	494.6	8.6	1800	Fang *et al*., 2012b
South LP	104.47	34.63	512	10.9	2436	Fang *et al*., 2010
South LP	106.53	35.52	503	8.8	2000	Song *et al*., 2013
South LP	106.53	35.52	511.2	8.6	1950	Hou *et al*., 2007
South LP	109.68	35.8	605	8.75	1100	Chen *et al*., 2014a
South LP	106.15	34.45			2100	Song *et al*., 2014
South LP	104.17	35.1				Fang *et al*., 2017
South LP	106.15	34.45			2080	Chen *et al*., 2014b
South LP	106.15	34.45			2080	Chen *et al*., 2013
South LP	108.9	34.3				Liu *et al*., 2012
South LP	110.08	34.48			2000	Chen *et al*., 2016
South LP	112.35	35.32	600	9.1	869	Cao *et al*., 2021
South LP	106.1	35.58	425	6.25	1753	Wang *et al*., 2009b

East LP	112.08	38.83	420	5	2400	Li *et al.*, 2016
East LP	113.45	37.4	520	10	1440	Cai & Liu, 2006
East LP	111.97	38.7			1800	Su *et al.*, 2012
East LP	113.73	39.67	377.2	6.1	1750	Cai *et al.*, 2013a
East LP	111.33	37.77	484.1	9.1	1800	Cai *et al.*, 2010
East LP	112.08	38.83	428		1900	Li *et al.*, 2013
East LP	111.28	40.78	400	6.75	1300	Liu *et al.*, 2016a
East LP	112.4	36.9			1450	Cai *et al.*, 2014
East LP	113.72	39.68			1750	Cai *et al.*, 2015
East LP	112.08	38.83			1800	Li *et al.*, 2015a
East LP	113.01	38.93			1700	Mei *et al.*, 2019
East LP	112.08	38.83			1900	Li *et al.*, 2015b
East LP	110.7	39.49	400	7	1347	Liang *et al.*, 2006
East LP	112.38	37.73			1300	Li *et al.*, 2016b
East LP	111.72	38.97			1745	Zhang *et al.*, 2018
East LP	110.4	39.3	343.4		1300	Yang *et al.*, 2013
East LP	110.42	39.29	400	7	1347	Liang *et al.*, 2004
East LP	111.33	37.77	463.8	9.7	1820	Cai & Liu, 2013
East LP	111.48	37.68	463.8	9.7	1690	Cai & Liu, 2013

Note: South LP, East LP and West LP indicate southern Loess Plateau (Qinling Mountains and its adjacent mountainous areas), eastern Loess Plateau (the Taihang and Lüliang Mountains) and western Loess Plateau (the northeastern margin of the Tibetan Plateau and the Henan Mountain)

References for Table S1

Cai, Q., Liu, Y., Lei, Y., Bao, G., and Sun, B. (2014). Reconstruction of the March-August PDSI since 1703 AD based on tree rings of Chinese pine (*Pinus tabulaeformis* Carr.) in the Lingkong Mountain, southeast Chinese loess Plateau. *Clim. Past*, 10, 509–521. doi:10.5194/cp-10-509-2014

Cai, Q. F., and Liu, Y. (2013). Climatic response of Chinese pine and PDSI variability in the middle Taihang Mountains, north China since 1873. *Trees Struct. Funct.*, 27, 419–427. doi:10.1007/s00468-012-0812-6

Cai, Q. F., and Liu, Y. (2013). Climatic response of three tree species growing at different elevations in the Luliang Mountains of Northern China. *Dendrochronologia*, 31, 311–317. doi:10.1016/j.dendro.2012.07.003

Cai, Q. F., Liu, Y., Bao, G. A., Lei, Y., and Sun, B. (2010). Tree-ring-based May-July mean temperature history for Luliang Mountains, China, since 1836. *Chin. Sci. Bull.*, 55(26), 3008–3014. doi:10.1007/s11434-010-3235-z

Cai, Q. F., Liu, Y., Liu, H., and Ren, J. L. (2015). Reconstruction of drought variability in North China and its association with sea surface temperature in the joining area of Asia and Indian-Pacific Ocean. *Palaeogeogr. Palaeoclimatol. Palaeoecol.*, 417, 554–560. doi:10.1016/j.palaeo.2014.10.021

Cai, Q. F., Liu, Y., Song, H. M., and Sun, J. Y. (2008). Tree-ring-based reconstruction of the April to September mean temperature since 1826 AD for north-central Shaanxi Province, China. *Sci. China Ser. D-Earth Sci.*, 51, 1099–1106. doi:10.1007/s11430-008-0084-6

Cai, Q. F., Liu, Y., and Tian, H. (2013a). A dendroclimatic reconstruction of May-June mean temperature variation in the Heng Mounatins, north China, since 1767 AD. *Quat. Int.*, 283, 3–10. doi:10.1016/j.quaint.2012.03.034

Cai, Q. F., Liu, Y., Yang, Y. K., Shi, J. F., Sun, J. Y., and Wang, L. (2005). The reconstruction of tree-ring chronology and early spring (from February to March) precipitation information in Huanglong region Shaanxi province. *Mar. Geol. Quat. Geol.*, 25, 133–139.

Cao, H. H., Zhao, X. E., Chen, F., Wang, S. J., and Liu, X. H. (2021). Reconstructing January-June precipitation in southeastern Shanxi over the past 296 years inferred from tree-ring records of *Pinus tabuliformis*. *Chin. J. Appl. Ecol.*, 32, 3618–3626. doi:10.13287/j.1001-9332.202110.018

Chen, F., and Yuan, Y. J. (2014b). May-June maximum temperature reconstruction from mean earlywood density in north central China and its linkages to the summer monsoon activities. *PLoS ONE*, 9, e107501.. doi:10.1371/journal.pone.0107501

Chen, F., Yuan, Y. J., Wei, W. S., Fan, Z. A., Yu, S. L., Zhang, T. W., et al. (2013). Reconstructed precipitation for the north-central China over the past 380 years and its linkages to East Asian summer monsoon variability. *Quat. Int.*, 283, 36–45. doi:10.1016/j.quaint.2012.05.047

Chen, F., Yuan, Y. J., Zhang, R. B., and Qin, L. (2014a). A tree-ring based drought reconstruction (AD 1760-2010) for the Loess Plateau and its possible driving mechanisms. *Glob. Planet. Change*, 122, 82−88. doi:10.1016/j.gloplacha.2014.08.008

Chen, F., Zhang, R. B., Wang, H. Q., Qin, L., and Yuan, Y. J. (2016). Updated precipitation reconstruction (AD 1482-2012) for Huashan, north-central China. *Theor. Appl. Climatol.*, 123, 723−732. doi:10.1007/s00704-015-1387-0

Fang, K. Y., Gou, X. H., Chen, F. H., D'Arrigo, R., and Li, J. B. (2010). Tree-ring based drought reconstruction for the Guiqing Mountain (China): linkages to the Indian and Pacific Oceans. *Int. J. Climatol.*, 30, 1137−1145. doi:10.1002/joc.1974

Fang, K. Y., Gou, X. H., Chen, F. H., Frank, D., Liu, C. Z., Li, J. B., and Kazmer, M. (2012a). Precipitation variability during the past 400 years in the Xiaolong Mountain (central China) inferred from tree rings. *Clim. Dyn.*, 39, 1697−1707. doi:10.1007/s00382-012-1371-7

Fang, K. Y., Gou, X. H., Chen, F. H., Liu, C. Z., Davi, N., Li, J. B., et al. (2012b). Tree-ring based reconstruction of drought variability (1615–2009) in the Kongtong Mountain area, northern China. *Glob. Planet. Change*, 80-81, 190−197. doi:10.1016/j.gloplacha.2011.10.009

Fang, K. Y., Guo, Z. T., Chen, D. L., Linderholm, H. W., Li, J. B., Zhou, F. F., et al. (2017). Drought variation of western Chinese Loess Plateau since 1568 and its linkages with droughts in western North America. *Clim. Dyn.*, 49, 3839–3850. doi:10.1007/s00382-017-3545-9

Gao, S. Y., Lu, R. J., Qiang, M. R., Hasi, E., Zhang, D. S., Chen, Y., and Xia, H. (2005). Reconstruction of precipitation in the last 140 years from tree ring at south margin of the Tengger Desert, China. *Chin. Sci. Bull.*, 50, 2487–2492. doi:10.1360/982005-363

Hou, Y., Wang, N. A., Li, G., and Zheng, F. (2007). Reconstruction of summer average temperature from tree-ring proxy data during 1751–2005 in Mt. Kongtong. *Adv. Clim. Chang.*, 3, 172–176.

Jia, F. F., Lu, R. J., Shang, Y., Tian, L. H., and Chen, Y. (2014). Influence of different detrending methods on climate signals from tree-ring width chronologies in Hasi Mountain, northwestern China. *Arid Land Geogr.*, 37(4), 676–683.

Kang, S. Y., Yang, B., and Qin, C. (2012). Recent tree-growth reduction in north central China as a combined result of a weakened monsoon and atmospheric oscillations. *Clim. Change*, 115, 519–536. doi:10.1007/s10584-012-0440-6

Li, J., Chen, F., Cook, E. R., Gou, X., and Zhang, Y. (2007). Drought reconstruction for North Central China from tree rings: the value of the Palmer drought severity index. *Int. J. Climatol.*, 27, 903–909. doi:10.1002/joc.1450

Li, Q., Liu, Y., Cai, Q. F., Sun, J. Y., Yi, L., Song, H. M., and Wang, L. (2006). Reconstruction of annual precipitation since 1686 AD from Ningwu region Shanxi province. *Quat. Sci.*, 26(6), 999–1006.

Li, Q., Liu, Y., Nakatsuka, T., Song, H., McCarroll, D., Yang, Y., and Qi, J. (2015b). The 225-year precipitation variability inferred from tree-ring records in Shanxi Province, the North China, and its teleconnection with Indian summer monsoon. *Glob. Planet. Change*, 132, 11–19. doi:10.1016/j.gloplacha.2015.06.005

Li, Q., Liu, Y., Song, H. M., Cai, Q. F., and Yang, Y. K. (2013). Long-term variation of temperature over North China and its links with large-scale atmospheric circulation. *Quat. Int.*, 283, 11–20. doi:10.1016/j.quaint.2012.03.017

Li, Q., Liu, Y., Song, H. M., Yang, Y. K., and Zhao, B. Y. (2015a). Divergence of tree-ring-based drought reconstruction between the individual sampling site and the Monsoon Asia Drought Atlas: an example from Guancen Mountain. *Sci. Bull.*, 60, 1688–1697. doi:10.1007/s11434-015-0889-6

Li, Y. J., Gou, X. H., and Fang, K. Y. (2012). Reconstruction of precipitation of previous August to current June during 1821–2008in the eastern Qilian Mountains. *J. Desert Res.*, 32, 1393–1401.

Li, Y. J., Wang, S. Y., Niu, J. J., Fang, K. Y., Chao, Y., Li, X. L., and Li, Y. H. (2016b). Tree-ring-based reconstruction of drought variability (1792-2011) in the middle reaches of the Fen River, North China. *Dendrochronologia*, 40, 1–11. doi:10.1016/j.dendro.2016.05.001

Liang, E. Y., Liu, X. H., Yuan, Y. J., Qin, N. S., Fang, X. Q., Huang, L., et al. (2006). The 1920S drought recorded by tree rings and historical documents in the semi-arid and arid areas of Northern China. *Clim. Change*, 79, 403–432. doi:10.1007/s10584-006-9082-x

Liang, E. Y., Shao, X. M., Huang, L., and Wang, L. L. (2004). The indication effect of drought disaster occurred at 1920s deduced from tree ring materials on the middle-western part of China. *The Advance of Natural Sciences*, 14, 469–474.

Liu, Y., Linderholm, H. W., Song, H., Cai, Q., Tian, Q., Sun, J., et al. (2009b). Temperature variations recorded in *Pinus tabulaeformis* tree rings from the southern and northern slopes of the central Qinling Mountains, central China. *Boreas*, 38, 285–291. doi:10.1111/j.1502-3885.2008.00065.x

Liu, Y., Tian, Q. H., Song, H. M., Sun, J. Y., Linderholm, H. W., Chen, D. L., et al. (2009a). Tree-ring width based May-June mean temperature reconstruction for Huashan Mountain since A. D. 1558 and 20th century warming. *Quat. Sci.*, 29, 888–895.

Liu, Y., Wang, R., Leavitt, S. W., Song, H., Linderholm, H. W., Li, Q., and An, Z. (2012). Individual and pooled tree-ring stable-carbon isotope series in Chinese pine from the Nan Wutai region, China: Common signal and climate relationships. *Chem. Geol.*, 330-331, 17–26. doi:10.1016/j.chemgeo.2012.08.008

Liu, Y., Zhang, X., Song, H., Cai, Q., Li, Q., Zhao, B., et al. (2016a). Tree-ring-width-based PDSI reconstruction for central Inner Mongolia, China over the past 333 years. *Clim. Dyn.*, 48, 867–879. doi:10.1007/s00382-016-3115-6

Lu, R. J., Gao, S. Y., Wang, Y. J., Ma, Y. Z., Qiang, M. R., and Zhang, D. S. (2013). Tree-ring based drought reconstruction at the northwestern margin of monsoon region of China since 1862. *Quat. Int.*, 283, 93–97. doi:10.1016/j.quaint.2012.03.051

Lu, R. J., Jia, F. F., Gao, S. Y., Shang, Y., and Chen, Y. (2016). Tree-ring reconstruction of January-March minimum temperatures since 1804 on Hasi Mountain, northwestern China. *J. Arid Environ.*, 127, 66–73. doi:10.1016/j.jaridenv.2015.10.020

Ma, L. M. (2003). The precipitation records from tree-ring latewood width in the Helan Mountain. *Mar. Geol. Quat. Geol.*, 23, 109–114. doi:10.16562/j.cnki.0256-1492.2003.04.016

Ma, Y. Y., Liu, Y., Song, H. M., Sun, J. Y., Lei, Y., and Wang, Y. C. (2015). A standardized precipitation evapotranspiration index reconstruction in the Taihe Mountains using tree-ring widths for the last 283 years. *PLoS ONE*, 10, e0133605. doi:10.1371/journal.pone.0133605

Mei, R., Song, H., Liu, Y., Payomrat, P., Cai, Q., Sun, C., and Fang, C. (2019). Tree-ring width-based precipitation reconstruction in Zhaogaoguan, China since 1805 AD. *Quat. Int.*, 510, 44–51. doi:10.1016/j.quaint.2018.12.015

Song, H. M., and Liu, Y. (2011). PDSI variations at Kongtong Mountain, China, inferred from a 283-year *Pinus tabulaeformis* ring width chronology. *J. Geophys. Res. Atmos.*, 116, D22115. doi:10.1029/2011jd016220

Song, H. M., Liu, Y., Li, Q., Gao, N., Ma, Y. Y.,and Zhang, Y. H. (2014). Tree-ring based May-July temperature reconstruction since AD 1630 on the western Loess Plateau, China. PloS ONE, 9, e93504. doi:10.1371/journal.pone.0093504

Song, H. M., Liu, Y., Li, Q., and Linderholm, H. (2013). Tree-ring derived temperature records in the central Loess Plateau, China.*Quat. Int.*, 283, 30–35. doi:10.1016/j.quaint.2012.03.033

Sun, J. Y., Liu, Y., Sun, B., and Wang, R. Y. (2012). Tree-ring based PDSI reconstruction since 1853 AD in the source of the Fenhe river basin, Shanxi province, China. *Sci. China Earth Sci.*, 55, 1847–1854. doi:10.1007/s11430-012-4369-4

Wang, M. M., Dai, J. H., Bai, J., and Cui, H. T. (2009b). Reconstruction of humidity changes from tree rings in Liupan Mountains area since 1900. *J. Palaeogeogr.*, 11, 355–360.

Wang, Y. J., Gao, S. Y., and Ma, Y. Z. (2010). Annual precipitation variation reconstructed by tree-ring width since AD 1899 in the west part of Hedong sandy area of Ningxia. *Arid Land Geogr.*, 33, 377–384.

Wang, Y. J., Lu, R. J., Ma, Y. Z., Sang, Y. L., Meng, H. W., and Gao, S. Y. (2013). Annual variation in PDSI since 1897 AD in the Tengger Desert, Inner Mongolia, China, as recorded by tree-ring data. *J. Arid Environ.*, 98, 20–26. doi:10.1016/j.jaridenv.2013.07.009

Wang, Y. J., Ma, Y. Z., Lu, R. J., Sang, Y. L., Meng, H. W., Hua, F. C., and Man, Z. H. (2009a). Reconstruction of mean temperatures of January to August since A. D. 1895 based on tree-ring data in the eastern part of the Oilian Mountains. *Quat. Sci.*, 29, 905–912.

Wang, Y. J., Ma, Y. Z., Zhen, Y. H., Lu, R. J., Sang, Y. L., and Meng, H. W. (2009c). Response of tree-ring width of *Pinus tabulaeformis* to climate factors in Luoshan Mountains of Ningxia. *J. Desert Res.*, 29, 971–976.

Yang, Y. K., Huang, Q., Liu, Y., Wang, W. K., Wang, Y. M., and Bai, T. (2013). Tree-ring density based precipitation reconstruction from June o October in central Ordos. *J. Northwest A&F Univ.*, 41, 96–109.

Zhang, Q., and Fang, O. Y. (2018). History of forest health from 1900 to 2012 in Xinzhou Prefecture, Shanxi Provine, China. *Acta Ecol. Sin.*, 38, 236–243. doi:10.5846/stxb20161222650

Zhang, Y. X., Wilmking, M., and Gou, X. H. (2009). Changing relationships between tree growth and climate in Northwest China. *Plant Ecol.*, 201, 39–50. doi:10.1007/s11258-008-9478-y

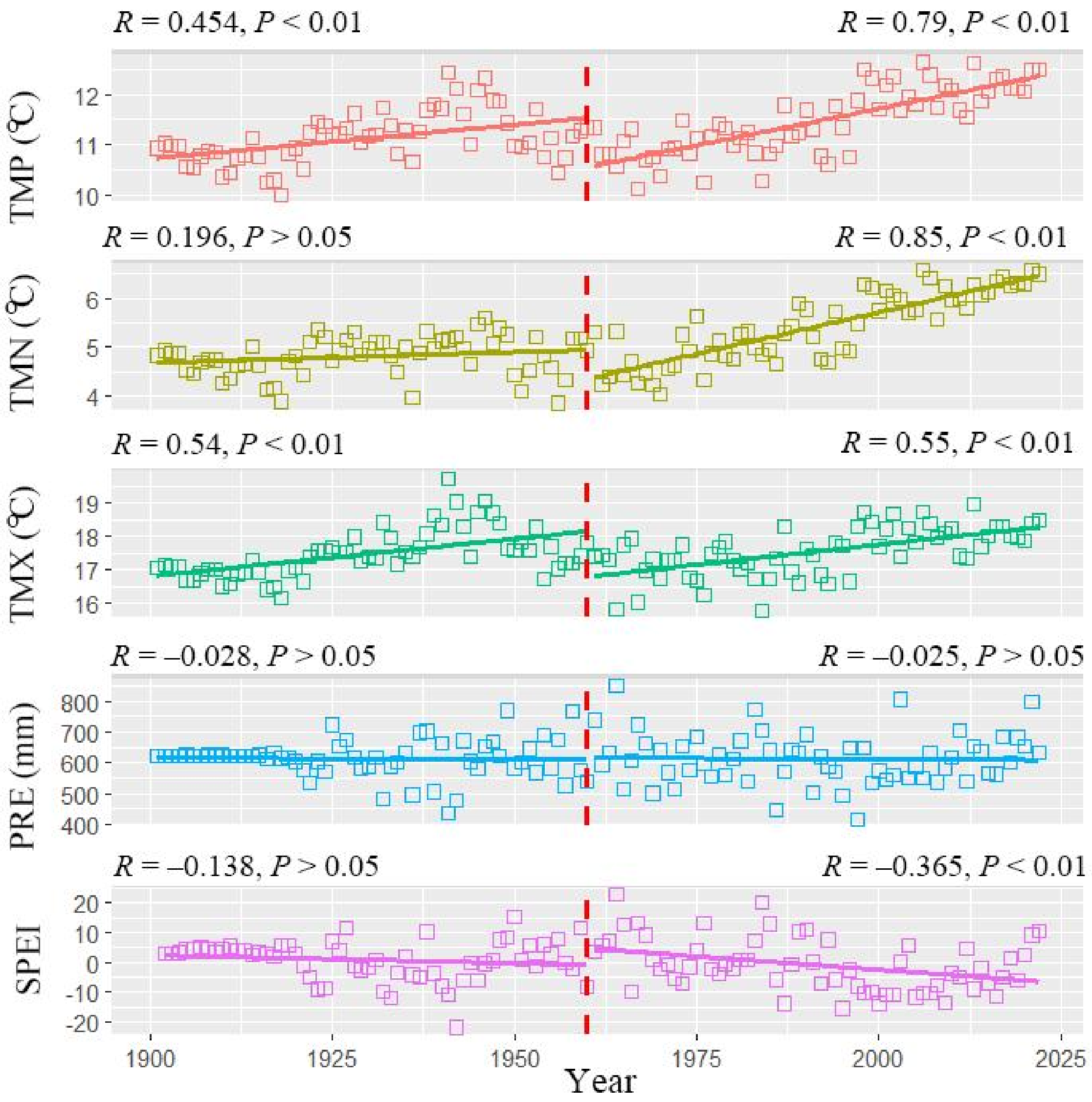

Figure S1 Comparison of changing trends during two distinct periods (1901–1960 and 1961–2012) for climatic variables in the southern Loess Plateau (the Qinling and surrounding mountainous areas).

Note: TMP, TMN, TMX, PRE, SPEI indicated mean temperature, minimum temperature, maximum temperature, total precipitation and standardized precipitation evapotranspiration index, respectively. *R* indicates the correlation coefficients between timeseries and climate data, and the red bold vertical dashed line indicates the year (1960) that separates the two analysis periods.

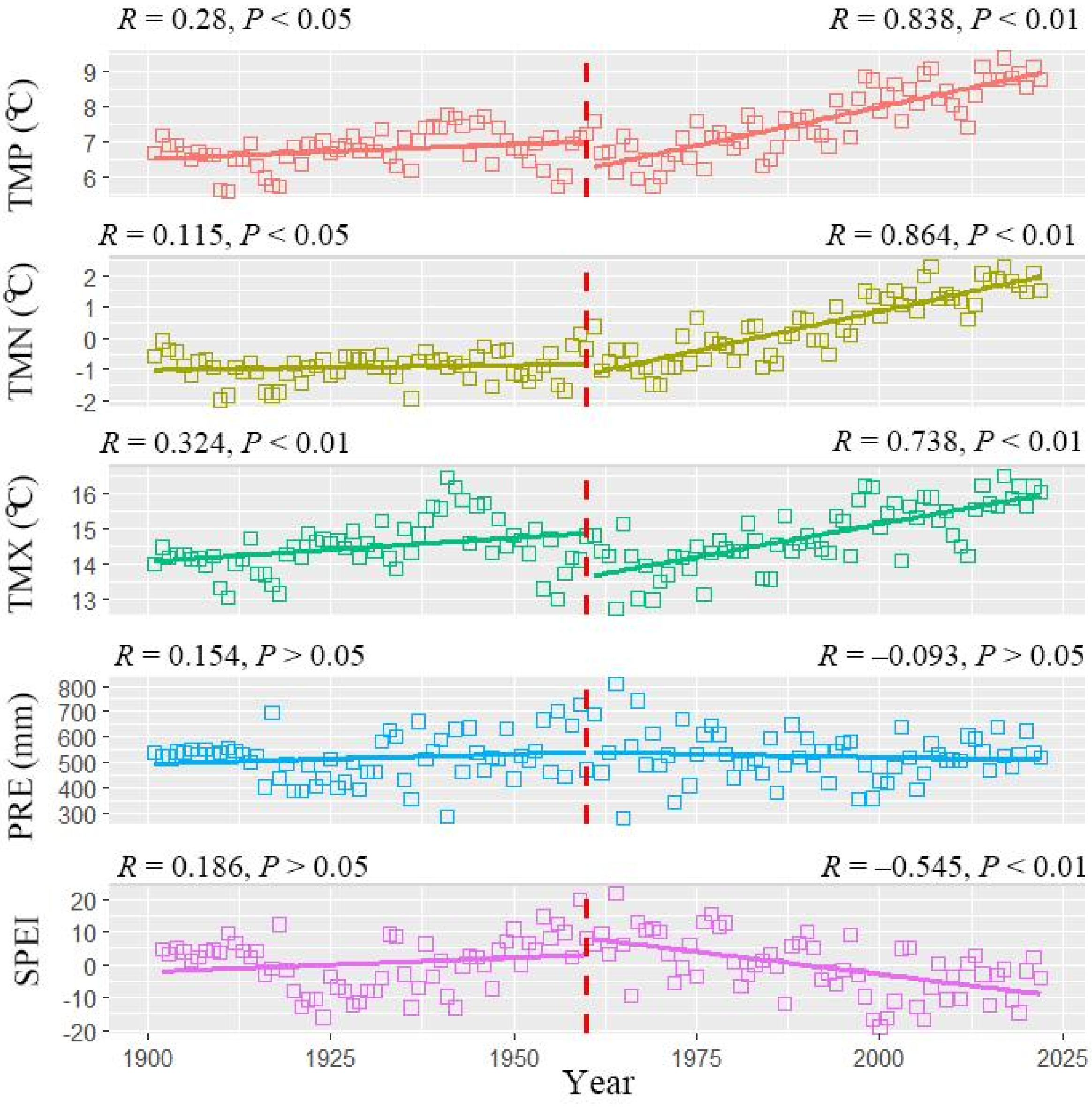

Figure S2 Comparison of changing trends during two distinct periods (1901–1960 and 1961–2012) for climatic variables in the eastern Loess Plateau (Taihang and Lüliang mountains).

Note: TMP, TMN, TMX, PRE, SPEI indicated mean temperature, minimum temperature, maximum temperature, total precipitation and standardized precipitation evapotranspiration index, respectively. *R* indicates the correlation coefficients between timeseries and climate data, and the red bold vertical dashed line indicates the year (1960) that separates the two analysis periods.

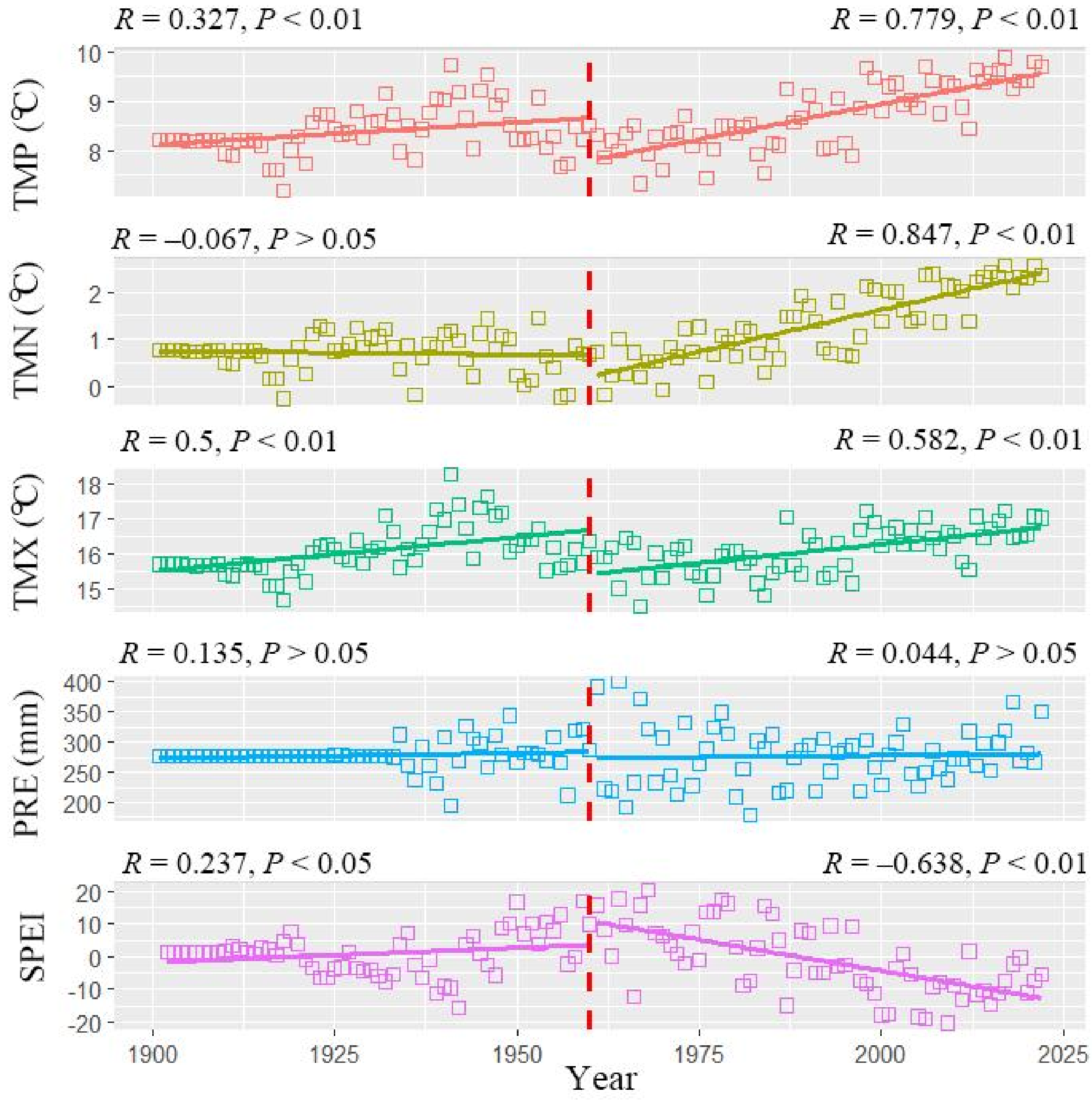

Figure S3 Comparison of changing trends during two distinct periods (1901–1960 and 1961–2012) for climatic variables in the western Loess Plateau (the northeastern Tibetan Plateau and Henan Mountain).

Note: TMP, TMN, TMX, PRE, SPEI indicated mean temperature, minimum temperature, maximum temperature, total precipitation and standardized precipitation evapotranspiration index, respectively. *R* indicates the correlation coefficients between timeseries and climate data, and the red bold vertical dashed line indicates the year (1960) that separates the two analysis periods.

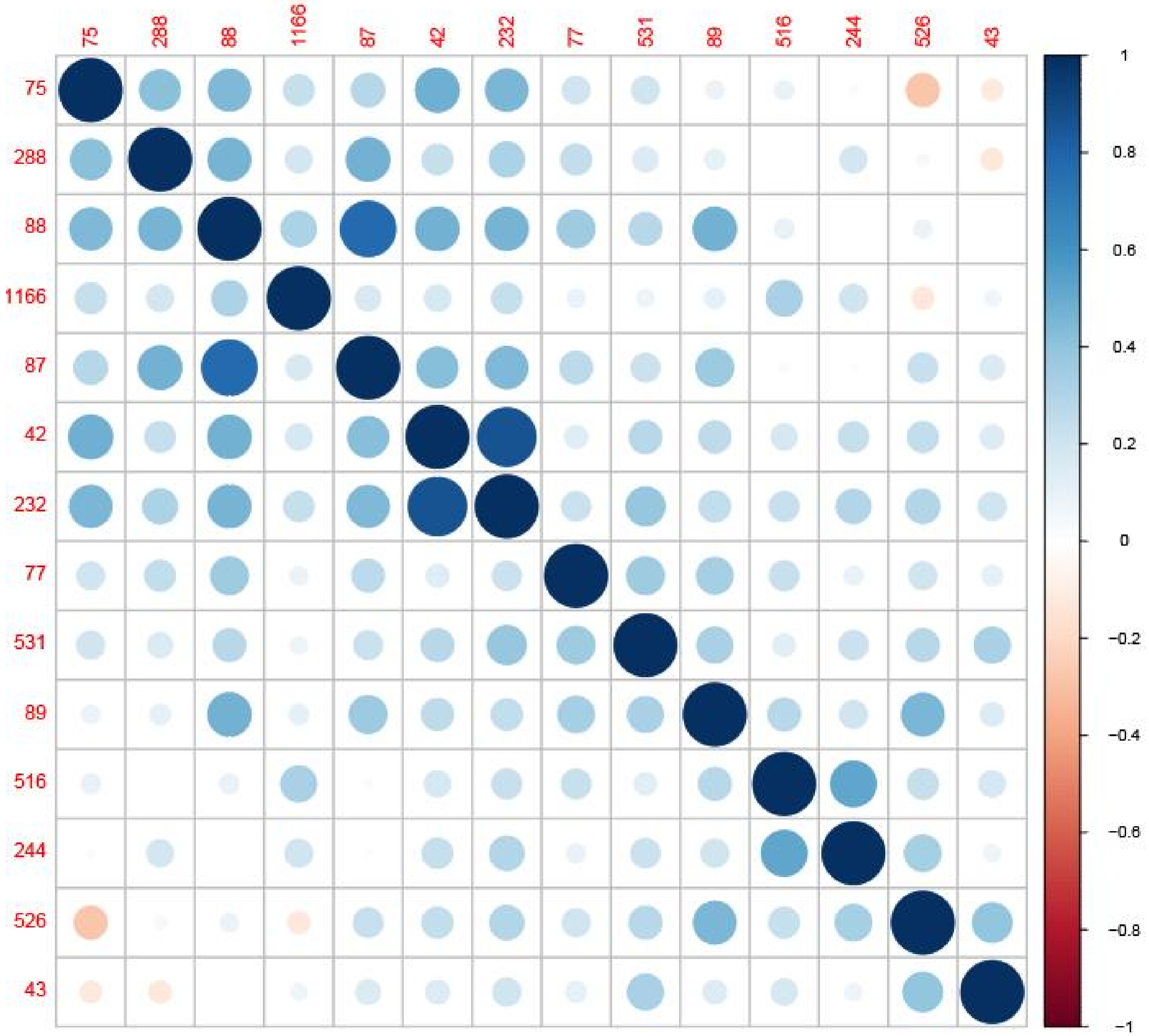

Figure S4 Correlation matrix of tree-ring chronologies (20 sites)for Chinese pine forests during the overlap period (1901–2003 period) in the southern Loess Plateau (the Qinling and surrounding mountainous areas).

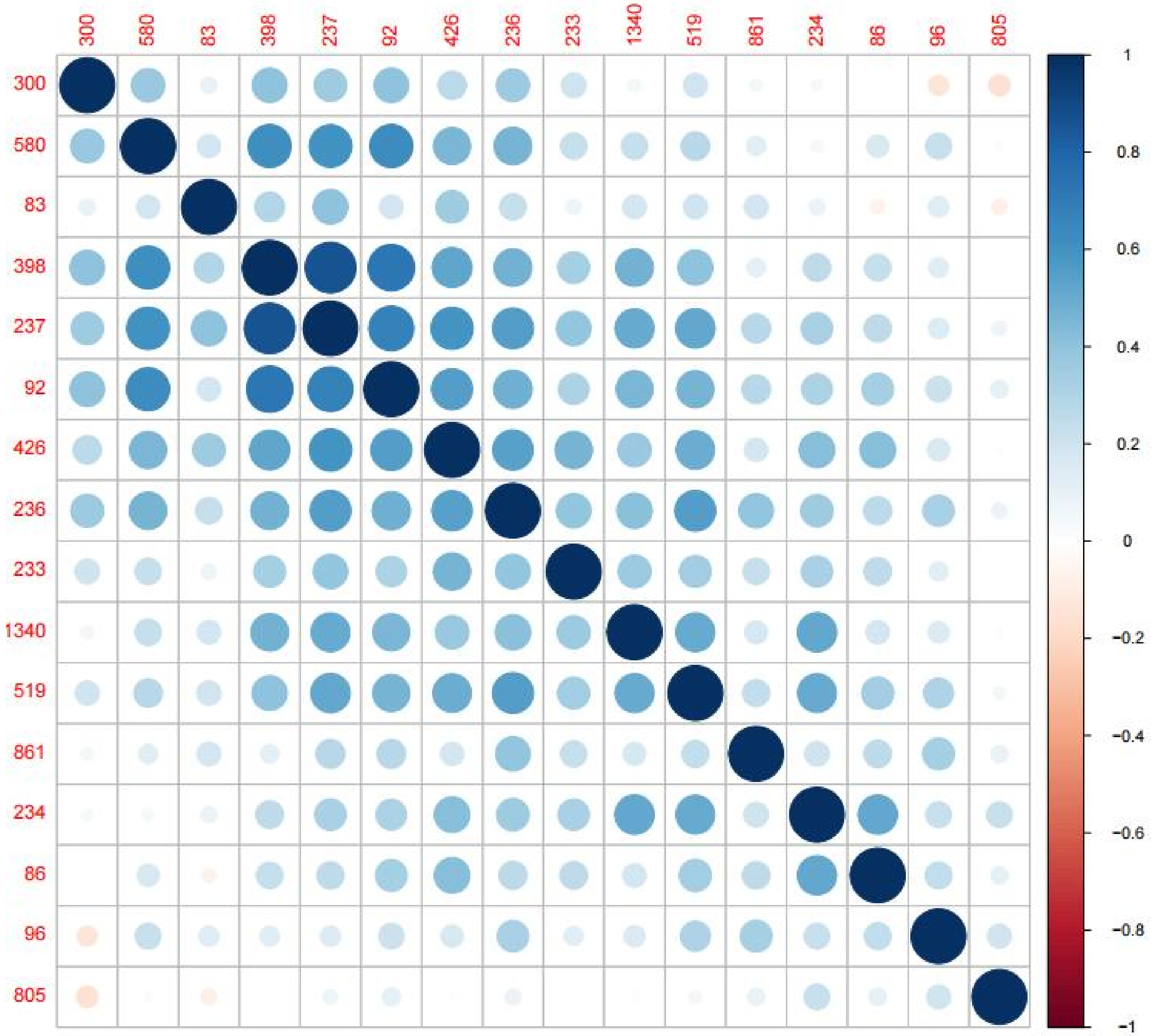

Figure S5 Correlation matrix of tree-ring chronologies (19 sites)for Chinese pine forests during the overlap period (1901–1999 period) in the eastern Loess Plateau (the Taihang and Lüliang mountains).

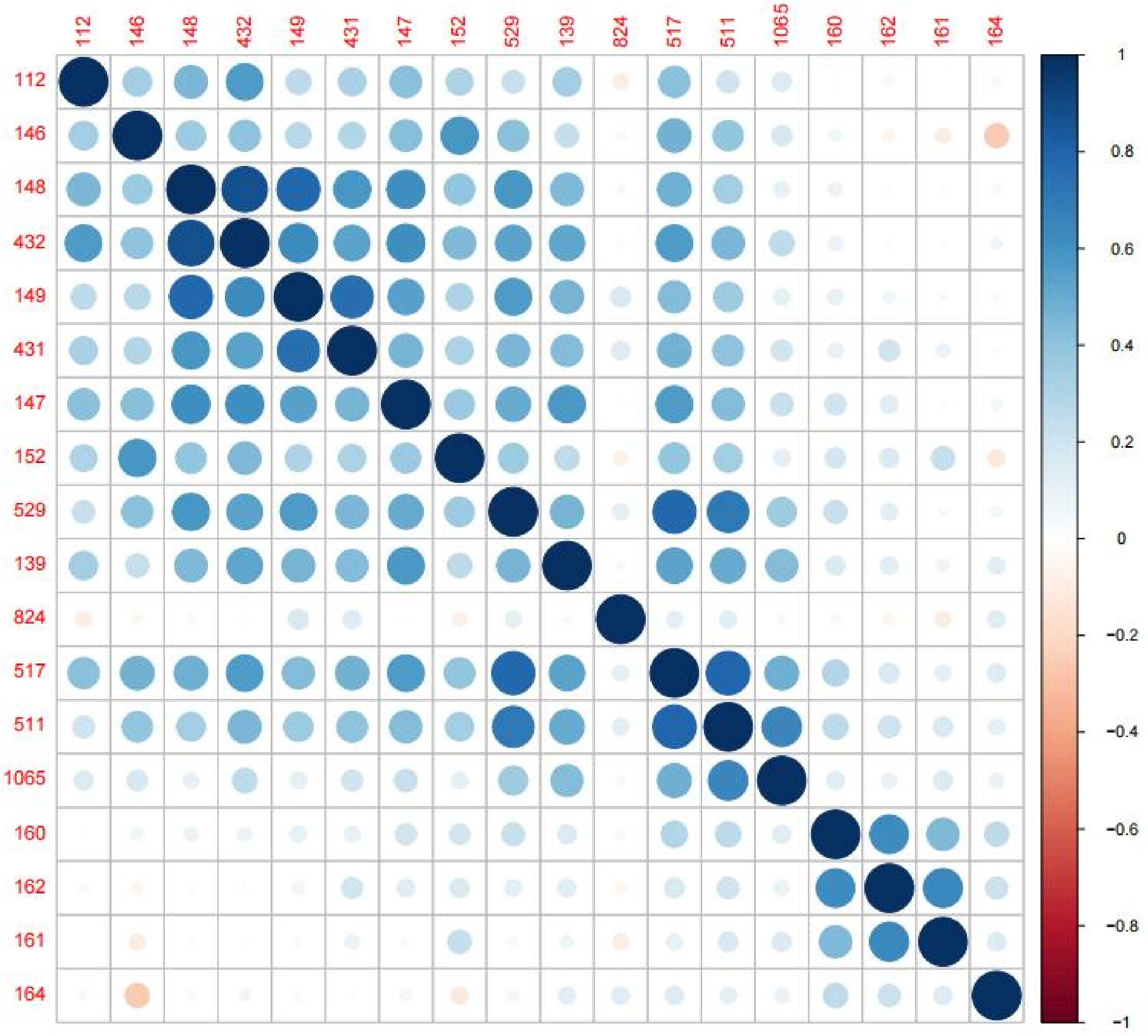

Figure S6 Correlation matrix of tree-ring chronologies (21 sites) for Chinese pine forests during the overlap period (1901–1998 period) in the western loess Plateau (the northeastern Tibetan Plateau and Helan Mountain).
